# Treatment trials in Niemann-Pick type C disease

**DOI:** 10.1007/s11011-021-00842-0

**Published:** 2021-10-01

**Authors:** Dominika Sitarska, Anna Tylki-Szymańska, Agnieszka Ługowska

**Affiliations:** 1 Department of Genetics, Institute of Psychiatry and Neurology, Al. Sobieskiego 9, 02-957 Warsaw, Poland; 2 Department of Pediatric Nutrition and Metabolic Diseases, The Children’s Memorial Health Institute, 04-730 Warsaw, Poland

**Keywords:** Niemann-Pick Type C disease, NPC, Therapy, Miglustat, HP-β-CD, Vorinostat, Arimoclomol, Gene Therapy

## Abstract

Niemann-Pick type C (NPC) disease is a genetically determined neurodegenerative metabolic disease. It belongs to the lysosomal storage diseases and its main cause is impaired cholesterol transport in late endosomes or lysosomes. It is an autosomal recessive inherited disease that results from mutations in the *NPC1* or *NPC2* genes. The treatment efforts are focused on the slowing its progression. The only registered drug, devoted for NPC patients is Miglustat. Effective treatment is still under development. NPC disease mainly affects the nervous system, and the crossing of the blood–brain barrier by medicines is still a challenge, therefore the combination therapies of several compounds are increasingly being worked on. The aim of this paper is to present the possibilities in treatment of Niemann-Pick type C disease. The discussed research results relate to animal studies.

## Introduction

Niemann-Pick type C disease is a neurodegenerative metabolic disease that belongs to the group of lysosomal storage diseases (LSD). It is a rare, inherited in an autosomal recessive manner condition, with an incidence of approximately 1.12:100,000 live births (Wassif et al. 2015). This disease is caused by pathogenic mutations in *NPC1,* located on chromosome 18q11.2 (Carstea et al. 1993) (OMIM #607,623), and *NPC2,* located on chromosome 14q24.3 (Naureckiene et al. 2000) (OMIM # 601,015), genes. The *NPC1* gene encodes a large, transmembrane 142 kDa protein located in the lysosomal membrane (Bauer et al. 2002). The *NPC2* gene encodes a small (MW = 16 kDa), soluble protein with high affinity to cholesterol, located inside late endosomes or lysosomes (Ko et al. 2003). Niemnn-Pick C is a neurovisceral disease. The phenotype of the disease is determined by the time of onset of the first symptoms. The most severe form is the neonatal, rapidly progressing cholestatic form. The clinical picture is dominated by liver involvement, (enlargement), along with the enlargement of spleen. Neurological symptoms manifest later on, although the life span is short. (Lipiński et al. 2018). This form is rare or underdiagnosed.

The second form is the early infantile form, with a severe course as well. The first noted symptom is hepatosplenomegaly, shortly after follow neurological symptoms as a regress in psychomotor development. Children affected with this form never learn to walk, later on their loss of motor skills and mental regression become more pronounced.

Late infantile and juvenile are the most common forms, the first symptoms are neurological symptoms appearing around 3–5 years of age. The most characteristic are gait problems, cognitive impairment, speech disturbance, movement—motor skills disorders, cerebellar ataxia and supra nuclear gaze palsy, seizures occur in most cases. Spleen enlargement is mild or moderate (Vanier 2013). The late onset form is slowly progressing, the neurological signs and symptoms are similar to those in juvenile form, spleen volume usually remains within normal values. This phenotype is characterized in one third of cases by psychotic presentation. This can be the only noticeable symptom for a long time (Di Lazzaro et al. 2016).

## Pathogenesis

For the development of an effective treatment, it is essential to know the molecular and biochemical bases of the disease. The pathomechanism of NPC is not yet fully understood. In human cells the cholesterol esters (CE) reach the interior of the lysosome by means of LDL. The lysosomal acid lipase cleaves free fatty acids (FFA) from the CE. FFAs cross the lysosome membrane into the cytoplasm. Free cholesterol (CH) is captured by the NPC2 protein and transported to the NPC1 protein localized in the lysosomal membrane. The NPC1 protein receives CH from NPC2 and transports it across the lysosomal membrane into the cytoplasm, where CH can then be reintroduced into the metabolic pathway. When NPC1 or NPC2 are not produced or the produced proteins are abnormal, it leads to lipid accumulation inside the lysosomes and the development of Niemann-Pick type C disease (Erickson 2013).

## Cholesterol lowering treatment

Considering that Niemann-Pick type C disease leads to the accumulation of cholesterol in lysosomes, the first idea to treat this disease was to try to decrease the quantity of the cellular cholesterol. Combined action of the medicines commonly used in the treatment of hypercholesterolaemia (cholestyramine, lovastatin and nicotinic acid) and low cholesterol diet reduced liver cholesterol level as well as serum total cholesterol, but the effects on neurological symptoms were not observed (Patterson et al. 1993). Other studies on animal models treated with Nifedipine (Calcium channel blocker) and Probucol (anti-hyperlipidemic drug) administered intraperitoneally showed a reduction in liver cholesterol levels without improvement in brain pathology. The authors concluded that it is possible that the pharmacological agents used do not penetrate the blood–brain barrier and, hence, do not affect the neurological progression of the disease (Erickson et al. 2000). These studies confirmed the ineffectiveness of cholesterol-lowering therapies, and it has even been speculated that this proves that cholesterol is not the major toxic metabolite in NPCs (Lloyd-Evans and Platt 2010).

Drug studies are conducted on animals, usually mice, whose main effectiveness criteria are animal survival and the assessment of the level of substrate accumulation in the brain.

## Miglustat

Miglustat (OGT 918, N-butyl-deoxynojirimycin) is an iminosugar that acts as an inhibitor of glucosylceramide synthase needed in the early stages of glycosphingolipid synthesis. This compound was initially approved by the Food and Drug Administration (FDA) for the treatment of Gaucher disease, which is also an LSD (Cox et al. 2000). The main advantage of Miglustat is its ability to cross the blood–brain barrier (BBB). Oral administration (1200 mg/kg daily) in *Npc1* mice reduced the accumulation of gangliosides in the brain, slowed the neurological progression of the disease, and increased lifespan by approximately 33% (Zervas et al. 2001). Subsequent studies on cats showed that a dose of 50 mg / kg per day was effective. Reduced accumulation of gangliosides in the brain was observed, delayed the onset of neurological symptoms and increased lifespan by approximately 74% (Zervas et al. 2001). Due to the promising results of animal studies, clinical trials were started in NPC patients in 2002 (clinicaltrials.gov). They showed neurological improvement or stabilization (e.g. dysphagia, supranuclear gaze palsy) (Pineda et al. 2019; Patterson et al. 2007; Wraith et al. 2010).

## Combination therapies with Miglustat

In 2009, the results of studies on the effects of anti-inflammatory therapy in the mouse model of NPC1 were published (Smith et al. 2009). Pre-symptomatic mice (3–5 weeks old), mice with the early symptoms of the disease (6–8 weeks old) and mice with the advanced disease (8–10 weeks old) were treated with i) ibuprofen, ii) Miglustat, and iii) the combined action of ibuprofen and Miglustat. Studies have shown that treating mice before the onset of symptoms and with advanced disease did not provide a clinical benefit on survival.

However, mice treated from the 6th week of age showed a statistically significant improvement in survival. Treatment with ibuprofen increased survival by an average of 1.67 weeks, treatment with Miglustat increased survival by 4.97 weeks, while the combination of Miglustat and ibuprofen resulted in an increased survival by 6.77 weeks (Smith et al. 2009). Williams et al. in 2014 published the results of a study on the therapeutic effects of Miglustat, Ibuprofen and Curcumin on *Npc1* mice. Mice were treated with Miglustat, ibuprofen, curcumin, curcumin + ibuprofen, Miglustat + curcumin, or a combination of these 3 substances. Miglustat and curcumin were given from the age of 3 weeks, while ibuprofen was given from 6 weeks of age because of proven toxicity with earlier dosing (Smith et al. 2009). The lifespan of untreated mice was approximately 10.5 weeks. Treatment with both ibuprofen and curcumin increased the life of the mice by about a week (9% and 11% respectively), while the combination of the 2 substances increased survival to 22%. Miglustat monotherapy resulted in a 5-week (46%) longer life. Combined therapy with Miglustat + curcumin brought the greatest benefits, increasing survival by about 8.8 weeks, and interestingly, it had better results than the combination of these 3 substances, which resulted in a longer life by about 7.7 weeks (Williams et al. 2014).

## HP-β-CD

HP-β-CD (2-Hydroxypropyl-β-cyclodextrin) is a cyclic oligosaccharide with a hydrophobic interior. It is initially absorbed into the endolysosome where it transports unesterified cholesterol to the cytosol and reduces its accumulation in the endolysosomes independently of NPC1 and NPC2 proteins. Unfortunately, HP-β-CD is not able to cross the BBB when administered systemically, possibly because of its large size. Studies in *Npc1*^−/−^ mice showed that intraperitoneal administration of HP-β-CD at a dose of 1500 mg/kg weekly reduced the accumulation of cholesterol in the liver (Camargo et al. 2001). On the other hand, the administration of single or multiple subcutaneous doses of 4000 mg/kg of 20% HP-β-CD solution reversed lysosomal cholesterol transport disturbances, significantly improved liver dysfunction, reduced lipid accumulation and neurodegeneration (Liu et al. 2009; Davidson et al. 2009). *Npc1* cats treated with subcutaneous HP-β-CD injections at 4000 and 8000 mg/kg also achieved some neurological improvement, however, at 8000 mg/kg, pulmonary toxicity or injection site intolerance developed (Vite et al. 2015). Much better results were obtained when *Npc1* cats were given HP-β-CD every 2 weeks at a dose of 120 mg intrathecally. This treatment reduced neurological dysfunction, lipid accumulation in neurons, and Purkinje cell death while avoiding pulmonary toxicity (Vite et al. 2015).

Animal studies have confirmed the importance of early intervention and starting treatment. In addition, a 6 times faster HP-β-CD clearance was found in older mice. To sum up, the highest effectiveness is demonstrated by administering the treatment simultaneously intraperitoneally / subcutaneously and intrathecally to the youngest animals. HP-β-CD is currently one of the most promising NPC therapeutic agents in clinical trials.

Other studies of Hoque et al. on HP-β-CD, HP-γ-CD, and their homolog 2-hydroxypropyl-α-cyclodextrin (HP-α-CD) on lipid accumulation in *Npc1*-null Chinese hamster ovary (CHO) cells shown that HP-β-CD and HP-γ-CD, unlike HP-α-CD, reduced intracellular free cholesterol levels and normalized the lysosome changes in *Npc1*-null cells but not in wild-type CHO cells. HP-β-CD and HP-γ-CD, unlike HP-α-CD, reduced the level of sphingomyelins in *Npc1*-null, but not wild-type, cells. 2-hydroxypropyl-γ-cyclodextrin appears to have therapeutic potential, but more research is needed (Hoque et al. 2020).

## Combination therapies with HP-β-CD

A 2009 study on the effectiveness of the combination therapy of Allopregnanolone with Miglustat brought unexpected results. The research consisted in administering Allopregnanolone dissolved in 2-hydroxypropyl-β-cyclodextrin (from 7 days of age) with Miglustat (from 10 days of age) to *Npc1* mice. Mice treated with combination therapy showed a delay of the onset of symptoms by approximately 2 weeks, and a sudden weight loss occurred 8 weeks later compared to untreated mice. Analysis of cholesterol accumulation by filipin staining revealed a decrease in cholesterol storage in neocortical neurons in treated animals. Purkinje cell viability was also assessed by calbindin staining. In mice undergoing combined therapy, Purkinje cells were found in all cerebellar lobules, while in untreated mice it was confined mainly to X lobules (Davidson et al. 2009). Further studies indicated a significantly higher benefit of HP-β-CD on the survival of mice than the main test substance, allopregnanolone. The obtained results confirmed that it was HP-β-CD in combination with Miglustat that increased the mice survival, and that allopregnanolone did not provide any additional benefits.

## Vorinostat

Vorinostat is an inhibitor of histone deacetylases that reduces the accumulation of lipids in the lysosomes of cultured skin fibroblasts of NPC patients. Munkacsi et al. performed in vitro studies of this compound in *Npc1* mouse models. The drug was administered intraperitoneally, 5 days a week at a dose of 150 mg / kg body weight. Disease progression was measured by gene expression, liver function and pathology, serum and tissue lipid levels, body weight, and the lifespan. Studies have shown reversal of liver dysfunction typical of NPC in treated mice, however, no positive effect on disease progression and animal survival. The concentration of Vorinostat in the brain was 100-fold lower than in the plasma. The metabolism of apolipoprotein B and the expression of main components of lipid homeostasis in primary hepatocytes from mutant mice were altered by treatment with Vorinostat. These results suggest that HDAC inhibitors are applicable in the treatment of visceral NPC disease, but improvement in BBB penetration is required to alleviate neurological symptoms (Munkacsi et al. 2017).

## Combination therapies with Vorinostat

Alam et al. conducted studies on a triple combination formulation (TCF) containing Vorinostat, HP-β-CD and polyethylene glycol (PEG) in an *Npc1* mouse model. Drug was administered intraperitoneally once a week at the dose of Vorinostat—50 mg/kg and HP-β-CD 2000 mg/kg. Studies have shown a positive effect of TCF therapy on histone acetylation in the brain of mice, neurites and Purkinje cells have been preserved. This delayed the symptoms of neurodegeneration and extended lifespan by about 5 months. The results suggest that the use of combination therapy increased the ability of vorinostat to cross the blood–brain barrier and did not cause a toxic effect even with long-term use (Alam et al. 2016).

## Arimoclomol

Arimoclomol is a hydroxamic acid derivative of bimoclomol that amplifies Heat Schock Protein (HSP) gene expression and facilitates the induction of HSPs, thus enhancing the endogenous cytoprotective mechanisms in the presence of cellular stress (Hargitai et al. 2003).

Arimoclomol acts as an amplifier of HSP expression in response to toxic misfolded proteins that aggregate during cellular stress. This is apparently achieved by stabilization of the active phosphorylated trimer of heat shock factor 1 (Hsf-1), which leads to amplified expression of a family of HSPs including HSP-70 and HSP-90. Arimoclomol may achieve this by increasing the level of phosphorylation of Hsf-1, thereby promoting a longer duration of Hsf-1 binding to heat shock response elements (HSE) on DNA (Hargitai et al. 2003; Lanka et al. 2009).

Arimoclomol is currently undergoing clinical trials to assess efficacy in the treatment of various neurodegenerative diseases (Kieran et al. 2004; Cudkowicz et al. 2008; Lanka et al. 2009; Parfitt et al. 2014; Kalmar et al. 2014; Benatar et al. 2009). Due to recent reports on the main role of HSP70 in the maturation of the NPC1 protein (Nakasone et al. 2014), as well as the positive effects of upregulation of heat shock proteins in vitro in NPC and other lysosomal diseases (Mu et al. 2008; Yang et al. 2014; Zampieri et al. 2012; Macías-Vidal et al. 2014), this therapy has become a promising research topic (Mengel et al. 2020) Kirkegaard et al. 2017 undertook in vitro testing of arimoclomol in primary fibroblasts from NPC patients and in vivo in the *Npc1* ^−/−^ mouse model.

Treatment of the NPC patient's fibroblasts significantly reduced the accumulation of non-esterified cholesterol in the lysosomes. The mouse *Npc1*^−/−^ model does not replicate the important missense mutations, which are the most common type of mutation in NPC, and therefore failed to assess heat shock protein induction during NPC1 folding and maturation. However, it confirmed the therapeutic benefits of HSP70 towards lysosomal instability.

Preliminary studies showed an improvement in ataxia and a 17% increase in survival in treated NPC mice. The optimal dose of the drug was found to be 10–30 mg/kg/ daily. Further studies showed that administration of the drug completely restored Hsp70 levels in the brain, but provided only a small increase in Hsp70 in the liver. The studies of Kikeegard et al. confirmed the neuroprotective effect of arimoclomol treatment in *Npc1*^−/−^ mice (Kirkegaard et al. 2017).

## Gene therapy

For many years, attempts have been made to identify the types of cells responsible for the pathology of the disease using the *Npc1* mouse model. Significant disease improvement was observed after the production of a functional Npc1 protein in *Npc1* mice using a prion (Loftus et al. 2002) or a transgene driven by a glial fibrillary acidic protein (GFAP) promoter-driven transgene (Zhang et al. 2008; Donohue et al. 2009; Kapur et al. 2009). Previous studies in mammals have shown that NPC1 is mainly localized in glia (Patel et al. 1999) and have suggested that both astrocytes and microglia mediate inflammation and neurodegeneration in *Npc1* mice (Baudry et al. 2003; Chen et al. 2007). Based on these studies, it can be concluded that the glial loss of NPC1 function is the direct cause of neuropathology. Although glial dysfunction is associated with the disease process, two independent studies using the mouse chimera or a conditional knock-out of *Npc1* have shown the cell-autonomous death of NPC1-deficient neurons (Elrickson et al. 2010).

The team of Lopez et al. hypothesized that the survival of neurons may also be cell-autonomous, in which case the function of NPC1 in neurons would be sufficient to improve neurological symptoms (Lopez et al. 2011). To test this hypothesis, Lopez et al. created a new NPC model that uses the powerful Tet system for induced and cell-specific gene expression in mice.

To identify the type of cell responsible for disease pathogenesis, an identifiable and functional NPC1 protein was produced in CNS neurons, astrocytes or visceral tissue from *Npc1*^−/−^ mice. The Tet system is a bitransgenic system requiring the use of reporter and driver transgene combination to gene expression control (Zhu et al. 2002). To precisely observe the localization and type specificity of NPC1 cells, a version of NPC1 with a fluorescent protein tag, NPC1-YFP, was required. Studies have shown that neuron-specific NPC1-YFP corrects CNS sterol accumulation in NPC mice, but does not prevent astrocyte cholesterol accumulation. In Niemann-Pick type C disease, the Purkinje cells (PN) located in the cerebellum are the most susceptible to NPC1 loss and are the first cells to degenerate (German et al. 2001), the greatest loss begins in the anterior part of the cerebellum (Sarna et al. 2003). In mice with a PNs-specific transgene, Purkinje cell survival was striking considering the age of the mice and the amount of NPC-YFP produced in the cerebellum (Lopez et al. 2011).

## Therapeutic perspectives: clinical Trials

The complexity of NPC disease has led to many different therapeutic attitudes. There are currently 15 clinical trials conducted worldwide on the treatment of Niemann-Pick type C disease, 10 of which concern HPβCD, under the name VTS-270, these trials are in Phase I, II and III. There are also 3 clinical trials on Miglustat, currently in Phase II, III and IV, and on Vorinostat (phase 1/2) and Arimoclomol (phase 2/3) (Data from 08/20/2020 clinicaltrials.gov). So far, the treatment of HPβCD is the most widely studied therapy with the best prognosis.

**In summary**, the treatment efforts applied in NPC were focused on i) decreasing the quantity of intra lysosomal free cholesterol, ii) reducing the synthesis of glucosylceramide by inhibiting the activity of its synthase, iii) restriction of inflammatory processes and immune system response, iv) strengthening the efflux of free cholesterol from the lysosomal compartment into cytosol, v) influencing the expression of genes needed to induce cell differentiation by inhibiting histone deacetylases (HDAC), vi) action of pharmacological chaperones to stimulate cellular protein repair pathway by activation of molecular chaperones such as heat shock proteins, vii) the development of gene therapy.

The research for effective treatment of NPC is still ongoing. So far, apart from Miglustat and Arimoclomol the clinical trials were focused on animal models. However, it should be emphasized that early introduction of medical intervention gives better results; thus the role of proper diagnostic process should not be underestimated.

## Data Availability

Not applicable.
